# Cognitive deficits in fibromyalgia syndrome are associated with pain responses to low intensity pressure stimulation

**DOI:** 10.1371/journal.pone.0201488

**Published:** 2018-08-01

**Authors:** Carmen M. Galvez-Sánchez, Cristina Muñoz Ladrón de Guevara, Casandra I. Montoro, María José Fernández-Serrano, Stefan Duschek, Gustavo A. Reyes del Paso

**Affiliations:** 1 Department of Psychology, University of Jaén, Jaén, Spain; 2 Department of Psychology, UMIT—University for Health Sciences Medical Informatics and Technology, Hall in Tirol, Austria; Taipei Veterans General Hospital, TAIWAN

## Abstract

**Background:**

Fibromyalgia syndrome (FMS) is a chronic disorder characterized by widespread musculoskeletal pain and diffuse tenderness, accompanied by complaints including morning stiffness, fatigue, insomnia and affective symptoms. In addition, affected patients frequently experience cognitive impairments such as concentration difficulties, forgetfulness or problems in planning and decision-making. These deficits are commonly ascribed to interference between nociceptive and cognitive processing.

**Method:**

The present study investigated the association of cognitive performance with (a) pain responses to low intensity pressure stimulation (0.45–2.25 kg/cm^2^), (b) responses to stronger (above-threshold) stimulation (2.70 kg/cm^2^), and (c) pain threshold and tolerance in 42 women with FMS. Tests of attention, memory, processing speed, and executive functions were applied.

**Results:**

While no significant correlations were seen for pain threshold and pain tolerance, inverse associations arose between pain intensity ratings during pressure stimulation and performance in all evaluated cognitive domains. The magnitude of the correlations increased with decreasing stimulus intensity.

**Conclusions:**

It may be concluded that pain experience during somatosensory stimulation of low intensity is more closely related to attention, memory and executive functions in FMS than the traditional measures of pain threshold and pain tolerance. Considering that pain responses to low intensity stimulation reflect the hyperalgesia and allodynia phenomena characterizing FMS, it may be hypothesized that central nervous pain sensitization is involved in cognitive impairments in the disorder.

## Introduction

Fibromyalgia syndrome (FMS) is a chronic disorder characterized by widespread musculoskeletal pain and diffuse tenderness. Accompanying symptoms include fatigue, insomnia, morning stiffness, depression and anxiety [1]. Cognitive deficits, such as forgetfulness, difficulties in concentration, mental slowness, language-related problems and reduced organization and planning abilities are also frequent in FMS [2–7]. According to patient reports, these deficits have a strong impact on psychosocial functioning and quality of life and therefore are among the most deleterious symptoms of the disorder [8–10].

Several factors have been implicated in the genesis of the cognitive impairments in FMS, such clinical pain, fatigue, insomnia and affective symptoms. The interference between central nervous nociceptive activity and cognitive processing seems to be the most important mediating mechanism. This is supported by numerous findings of close associations between cognitive deficits and the severity of clinical pain in FMS [5, 7, 11–15]. It has been argued that in FMS, exaggerated central nervous pain processing interferes with cognition, because it requires enhanced neural resources in brain areas that are involved in attention, memory and higher cognitive processes, as well as in nociception [7, 13–15].

Most studies demonstrating correlations between pain perception and cognitive performance in FMS used self-report measures of clinical pain [5, 7, 16–21]. Further studies have reported associations between cognition and pain intensity ratings on experimentally induced or behavioral pain indices, such as pain threshold and tolerance [13, 21–26].

Central-nervous sensitization to pain is one of the most important factors in the pathogenesis of FMS [23, 27–31]. Pain sensitization causes the typical hyperalgesia and allodynia that constitute the main symptoms of the disorder. It may be hypothesized that subjectively reported pain during somatosensory stimulation of low intensity (i.e., allodynia) would characterize central pain sensitization better than responses to higher pain intensity stimuli or traditional indices of pain threshold and tolerance. Consistent with this assumption, it has been shown that pain responses to physical stimulation intensities near the pain threshold allow for optimal differentiation between chronic pain patients and healthy individuals, and that these responses are closely associated with self-report measures of clinical pain severity, pain catastrophizing, depression or anxiety [28–33]. Increased processing of below-threshold pain stimuli in FMS is also reflected by hypervigilance to painful stimuli [13, 34], and perceptual amplification of auditory, electrical and tactile stimuli [35].

The aim of this study was to investigate the relationship between the cognitive impairments seen in FMS (in the domains of attention, visual and verbal memory, processing speed, cognitive flexibility, planning and organizational abilities) and (a) pain responses to pressure stimulation of varying intensities (including below-threshold intensities), and (b) pain threshold and pain tolerance as traditional behavioral measures of pain sensitivity. As we hypothesized that responses to low intensity stimulation reflect central-nervous sensitization and perceptual amplification better than responses to higher intensities, we expected that cognitive performance would be more closely related to pain reports during low intensity stimulation than (a) to those during stronger stimulation and (b) to pain threshold and tolerance.

## Materials and methods

### Participants

Forty-two women with FMS, recruited from the Fibromyalgia Association of Jaén (Spain), participated in the study. Mean age was 50.33 ± 8.76 years and mean body mass index (BMI) 27.32 ± 4.94 kg/cm^2^. All patients were examined by a rheumatologist and met the Fibromyalgia´ diagnostic criteria of the American Colleague of Rheumatology (ACR 2010) [1]. For control purposes, 30 healthy women, who did not differ significantly from patients in terms of age (47.50 ± 7.60 years, t(70) = 1.43, p = .41) or BMI (25.65 ± 3.46 kg/cm^2^, t(70) = 1.60, p = .25) also participated in the study. Exclusion criteria for both groups included the presence of metabolic abnormalities, neurological disorders, drug abuse, or severe somatic (e.g., cancer) or psychiatric (e.g., psychotic) diseases. All participants were right-handed. The study is part of a larger project on cognition in FMS [2, 6].

### Cognitive assessment

The following cognitive tests were used:

- The Spanish version of the *Rey-Osterrieth Complex Figure Test* (ROCF) [36] was used to measure visuospatial memory performance. In the test, an abstract figure comprising 18 parts is presented and the participant has to copy it on a sheet. Thirty minutes after, he/she is asked to reproduce the figure from memory. The total number of correctly reproduced parts and execution time during both conditions (i.e., copy and reproduction) were taken as performance indices.
- The *Verbal Learning Test* (TAVEC) [37] was used to assess verbal memory function. Firstly, a list of 16 words (shopping list) is read to the participant five times (List A); the participant has to reproduce as many words as possible immediately after each trial (immediate free recall). Thereafter, another list is read once (List B) and then has to be reproduced (interference control condition). Following a 20-minute break, a list of 44 words is read, which includes all words of List A, some words of List B, and some distractor words not included in either list. The participant has to decide whether or not each of these words belongs to List A (recognition task). Performance parameters comprised the number of correct responses during immediate free recall (List A and List B), false-positive (FP) responses and bias during the recognition task. Bias indicated the tendency during the recognition task to respond yes or no, and was computed according to the formula [37]
  $$\mathrm{Bias}=\left(\frac{\mathrm{FP}‑\mathrm{Omissions}}{\mathrm{FP}+\mathrm{Omissions}}\right)$$
- The Spanish adaptation of the *Zoo Map Task* (ZMT) from the Behavioural Assessment of the Dysexecutive Syndrome [38] was used to assess planning and organizational abilities. Version 2 of the task, in which participants are required to plan a route to visit 6 of 12 possible locations following an externally imposed strategy, was used. Execution time and correct answers were used as performance indices.
- The Spanish version of the *Trail Making Test* (TMT) [39] was used to evaluate processing speed, attention and cognitive flexibility. In the test, visual targets (numbers, letters) are presented on sheets of paper. It includes the following tasks, all of which have to be executed as fast as possible: (1) number sequence (connect the numbers 1 to 16 in sequential order), (2) letter sequence (connect the letters A to P in alphabetic order) and (3) switching (connect numbers and letters in alternating order, i.e. 1, A, 2, B etc.). Execution time of the tasks (in seconds) was the dependent variable.

### Pain stimulation and quantification

Pain was evoked using a wireless pressure algometer (Tracker Freedom, JTECH Medical, Lawndale, USA) with a stimulation surface area of 1 cm^2^. A computer allowed for control of the rate of increase in pressure (kg/s). The algometer was inserted in a screw-piston specifically designed to fix and press the fingernails. Pain pressure was delivered to the nail of the index finger of the left hand. Pain threshold (the pressure at which the participant started to feel pain) and tolerance (the maximum tolerated pressure) were evaluated at a rate of increase in pressure of 1 kg/s. Subjective pain intensity was assessed using a 10-cm visual analogue scale (VAS), for the question “How strong was the pain?”, ranging from 0 (not at all) to 10 (extremely).

### Procedure

The study was conducted in two sessions performed on separate days. Participants were asked not to consume non-opioids analgesic and/or opioids drugs for 24 hours before the study. No instructions were provided regarding anxiolytics and antidepressants, which were consumed as habitual. During the first session, clinical histories, medication use, and socio-demographic data were recorded. It was confirmed that there were no violations of the exclusion criteria. Then, participants were informed about the concepts of pain threshold and tolerance and the use of the VAS. To familiarize participants with the method, seven pressure stimuli of 5 s duration (with 20 s inter-trial intervals) were applied in the following intensity sequence: 1.35, 4.50, 0.90, 2.70, 0.45, 1.80, and 3.60 kg/cm^2^. Thereafter, pain threshold and tolerance were measured. Finally, six pressure stimuli of 5 s duration (20 s inter-stimulus intervals) were presented in ascending order: 0.45, 0.90, 1.35, 1.80, 2.25 and 2.70 kg/cm^2^ (increases in 0.45 kg/cm^2^ intervals). Immediately after each of these six pressure stimuli subjective pain intensity was assessed by the VAS. If an individual’s tolerance level was reached before the 2.70 kg/cm^2^ stimulation, the sequence was interrupted.

Two days later, during the second session, the neuropsychological tests were administered in the following order: ROCF (copy), ZMT, ROCF (reproduction), TAVEC (free recall), TMT, and TAVEC (recognition). The tests were presented in this sequence to avoid interference between different cognitive domains, especially between visual and verbal memory tasks. Between each test, participants had a 5-minute break. The study protocol was approved by the Ethics Committee for Human Research of the University of Jaén and all participants provided written informed consent.

### Statistical analysis

Associations between cognitive test parameters, pain threshold and tolerance, and VAS ratings of pain intensity were quantified using Pearson correlations. Differences between correlation coefficients were tested for significance using Fisher´s Z statistic. Group comparisons of pain threshold and pain tolerance were performed using Student’s t-tests for independent samples. VAS scores were analyzed via repeated measures ANOVA with group (FMS patients vs. healthy women) as the between-subject factor and stimulation intensity (five pressure levels) as the repeated-measures factor. The last value of the ascending series (2.70 kg/cm^2^) was not included in this analysis, as five patients had reached their tolerance level before this condition. The Greenhouse-Geisser correction was applied for adjustment of degrees of freedom. Results are reported with the original degrees of freedom and corrected p-values. The significance level was set at .05 in all analyses.

## Results

**Fig 1:**
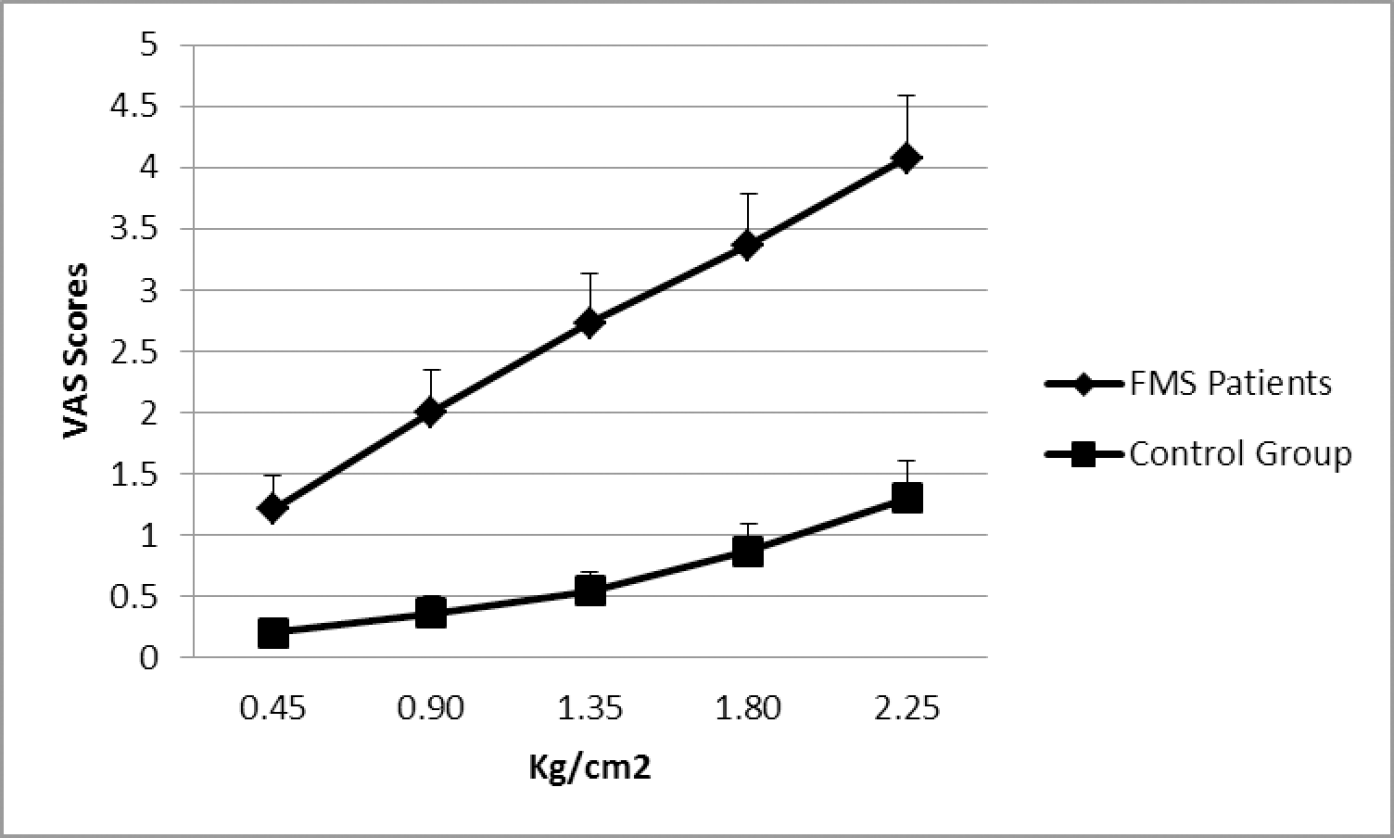
Pain intensity ratings (VAS scores) during the ascending pain stimulation series in FMS patients and healthy controls.

FMS patients, as compared to healthy women, exhibited a lower pain threshold (1.78 ± .93 vs. 4.34 ± 2.44 kg/cm^2^ for patients and controls, respectively, *t* = -5.35, *p* < .0001) and lower pain tolerance (4.11 ± 1.68 vs. 8.26 ± 2.54 kg/cm^2^, for patients and controls, respectively, *t* = -7.56, *p* < .0001). Subjective pain intensity ratings (VAS) increased with increasing pressure (repeated-measures effect: (F(4, 280) = 49.61, p < .0001, $\eta_{\rho }^{2}$ = .42) but as a function of group (interaction group x pressure intensity: (F(4, 280) = 22.96, p < .0001, $\eta_{\rho }^{2}$ = .13). The increase in VAS ratings was greater in FMS patients (F(4, 164) = 45.07, p < .0001, $\eta_{\rho }^{2}$ = .52) than in healthy women (F(4, 116) = 14.76, p < .0001, $\eta_{\rho }^{2}$ = .34) (see Fig 1). Moreover, VAS scores were higher overall in patients than in the healthy group (group effect: F(1, 70) = 18.58, p < .0001, $\eta_{\rho }^{2}$ = .21).

Table 1 includes the descriptive statistics of the cognitive test parameters in the FMS group. Group differences in these parameters have been reported elsewhere [2]. Table 2 presents the correlations between test performance and pain indices in the FMS sample. None of the correlations reached significance for pain threshold or tolerance. In contrast, several significant correlations arose for VAS scores, in particular those obtained during lower pressure intensities. For the ROCF, VAS scores correlated negatively with the number of correct marks in the copy (0.45, 0.90, 1.35, 1.80, 2.25 kg/cm^2^) and reproduction conditions (0.90, 1.80 kg/cm^2^), and positively with execution time in the copy condition (0.45, 0.90, 1.35 kg/cm^2^). Furthermore, VAS scores correlated positively with execution time (0.45, 0.90, 1.35, 1.80 kg/cm^2^) and negatively with correct responses (0.45, 0.90, 1.80 kg/cm^2^) in the ZMT. Concerning the TAVEC, VAS scores were positively associated with the number of FP responses (0.45, 0.90, 1.35, 1.80 kg/cm^2^) and recognition bias (0.45, 0.90, 1.35, 1.80, 2.25 kg/cm^2^). Finally, VAS scores correlated positively with execution time in the number sequence (0.45, 0.90, 1.35, 1.80, 2.25 kg/cm^2^), letter sequence (0.45, 0.90, 1.35 kg/cm^2^) and switching conditions (0.45, 0.90, 1.35 kg/cm^2^) of the TMT.

**Table 1:** Means (±SD) of cognitive test parameters in the FMS group.

	Cognitive variable	Mean±SD
**ROCF**	Copy: total correct marks	32.26 ± 3.40
	Reproduction: total correct marks	15.69 ± 5.75
	Copy: execution time	211.02 ± 94.17
	Reproduction: execution time	143.26 ± 55.25
**ZMT**	Correct responses (version 2)	6.90 ± 1.38
	Execution time (version 2)	126.81 ± 87.07
**TAVEC**	List A (immediate free recall)	45.12 ± 13.09
	List B (interference control)	4.62 ± 2.17
	Recognition false positives	4.33 ± 4.77
	Recognition bias	0.04 ± 0.40
**TMT**	Number sequence (execution time)	67.12 ± 47.02
	Letter sequence (execution time)	84.33 ± 55.97
	Switching (execution time)	179.60 ± 94.91

Note: Visual-spatial memory: *Rey-Osterrieth Complex Figure* (ROCF). Organization and decision-making process: Z*oo Map Test* (ZMT). Verbal memory: *Verbal Learning Test (TAVEC)*. Processing speed, attention and cognitive flexibility: *Trail Making Test* (TMT). All execution times are indicated in s.

**Table 2:** Correlations of cognitive test parameters with VAS scores, pain threshold and pain tolerance.

		VAS scores Pain Pain threshold tolerance						
		0.45 kg/cm^2^	0.90 kg/cm^2^	1.35 kg/cm^2^	1.80 kg/cm^2^	2.25 kg/cm^2^		
**ROCF**	Copy: total correct marks	-.14	-.22	-.22	-.22	-.23	-.03	< .01
	Reproduction: total correct marks	-.63* ^a^ ^b^	-.63* ^a^ ^b^ ^c^	-.58* ^a^ ^b^ ^c^	-.48* ^a^	-.46* ^a^	.15	.22
	Copy: execution time	.49* ^a^ ^b^ ^c^	.43* ^a^ ^b^ ^c^	.38^+^ ^a^ ^c^	.30 ^a^ ^c^	.22	.01	-.14
	Reproduction: execution time	.29	.31^+^	.29	.32^+^	.28	.12	.21
**ZMT**	Correct responses (version 2)	-.35^+^	-.35^+^ ^b^	-.29	-.31^+^	-.27	.15	.06
	Execution time (version 2)	.53* ^a^ ^b^ ^c^	.46* ^a^ ^b^ ^c^	.40* ^a^ ^b^	.39* ^a^ ^b^ ^c^	.31^+^	-.08	-.11
**TAVEC**	List A (immediate free recall)	-.12	-.03	.00	.03	.07	.08	.05
	List B (interference control)	-.24	-.21	-.18	-.10	-.08	.07	.09
	Recognition false positives	.44* ^a^ ^b^	.37^+^ ^b^	.38^+^ ^b^ ^c^	.34^+^ ^b^ ^c^	.25	-.11	.00
	Recognition bias	.54* ^a^ ^b^	.46*	.49* ^a^ ^b^	.50* ^a^ ^b^	.48* ^a^ ^b^	-.22	-.22
**TMT**	Number sequence (execution time)	.61* ^a^ ^b^ ^c^	.52* ^a^ ^b^ ^c^	.45* ^a^ ^b^ ^c^	.38^+^ ^a^ ^b^	.32^+^ ^a^ ^b^	-.02	-.04
	Letter sequence (execution time)	.54* ^a^ ^b^ ^c^	.41* ^a^ ^b^ ^c^	.33^+^ ^a^ ^b^ ^c^	.27 ^c^	.20	-.03	-.04
	Switching (execution time)	.48* ^a^ ^b^ ^c^	.40* ^a^ ^c^	.31^+^ ^c^	.22	.16	-.03	-.17

Note: Significant differences between correlation coefficients (Fisher´s Z statistic) are indicated as follows

^a^ for difference with pain threshold

^b^ for difference with pain tolerance

^c^ for difference with 2.25 kg condition

^+^ for p < 0.05

* for p < 0.01.

Correlations were higher overall for the VAS scores pertaining to the lower pressure conditions versus those of the 2.25 kg/cm^2^ condition and pain threshold and tolerance. Significant differences between the coefficients of the VAS scores and those of pain threshold and tolerance (Fisher´s Z test) were seen for correct marks in the reproduction condition and execution time in the copy condition of the ROCF, execution time of the ZMT, FPs and recognition bias of the TAVEC, and execution times in the number sequence, letter sequence and switching conditions of the TMT (see Table 2). Significant differences between coefficients of the 2.25 kg/cm^2^ pressure and those of the lower pressures (0.45, 0.90, 1.35, 1.80 kg/cm^2^) were found for correct marks in the reproduction condition and execution time in the copy condition of the ROCF, execution time of the ZMT, FPs in the TAVEC and execution times in the number sequence, letter sequence and switching conditions of the TMT (see Table 2).

**Fig 2:**
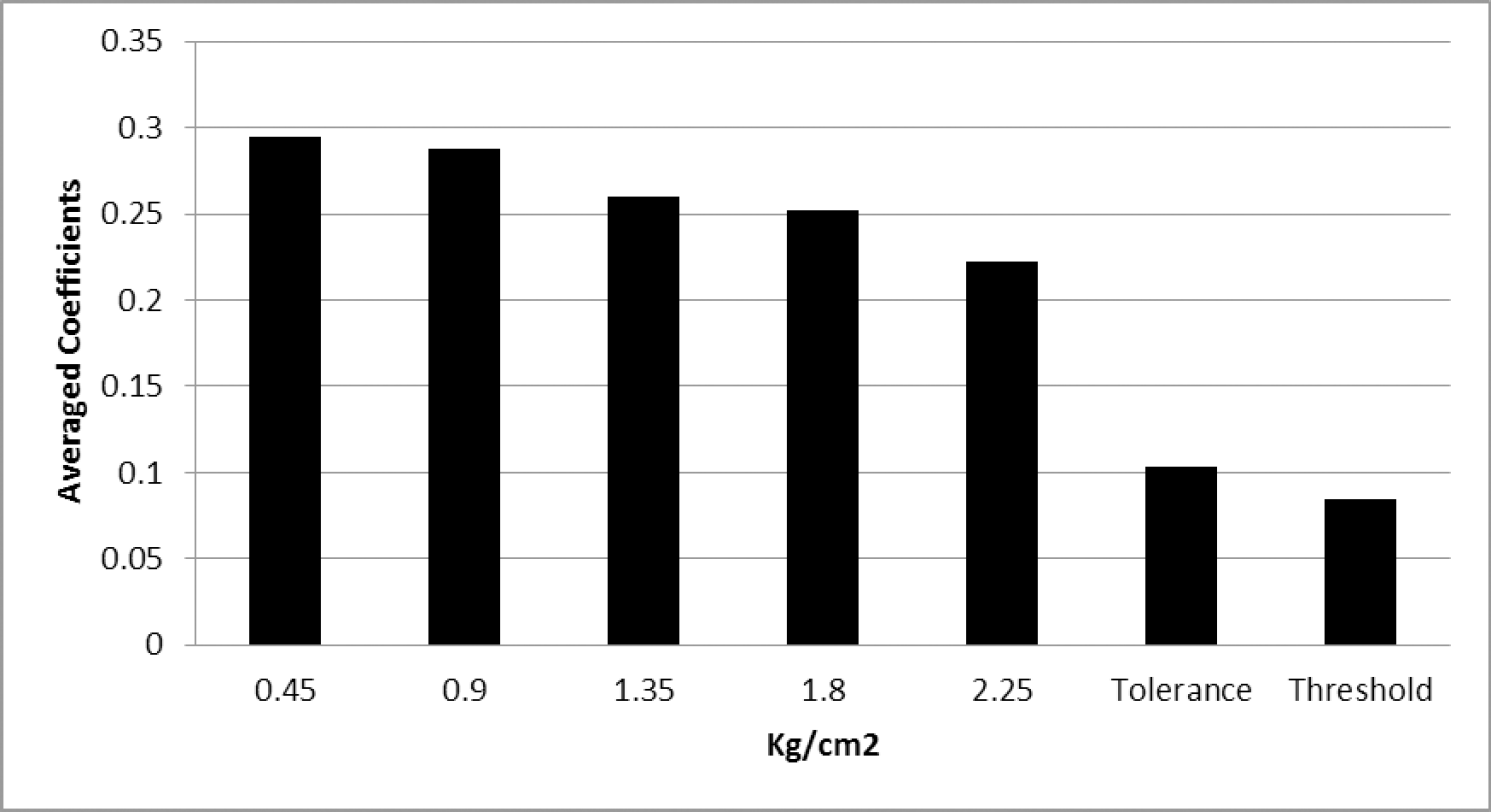
Averaged correlations (from absolute values) for all neuropsychological variables and pain reports as a function of pain pressure intensity.

Fig 2 displays the magnitudes of the correlations (absolute values) between pain indices and cognitive performance averaged across all test parameters. The correlations were lowest for pain threshold and pain tolerance. Higher correlations were obtained for the VAS scores; their size progressively decreased with increasing intensity of pressure stimulation. As indicated in Table 2, the number of significant correlations with performance indices also increased with decreasing stimulation pressure intensity, i.e., 4, 7, 8, 10, 9 significant coefficients for the 2.25, 1.80, 1.35, 0.90, and 0.45 kg/cm^2^ conditions.

## Discussion

In accordance with previous findings, our study demonstrated lower pain thresholds and pain tolerance in FMS patients than healthy individuals [e.g., 7, 28, 29]. Patients also reported higher pain intensity during pressure stimulation, where the slope of the increase in pain intensity across series of ascending pressures was greater in patients than controls. FMS patients rated very low pressure intensities as painful, reflecting the phenomenon of allodynia. These results reflect the hyperalgesia that characterizes the disorder and are congruent with the central pain sensitization hypothesis of FMS [28, 30, 40–42].

At first sight, it may seem surprising that FMS patients evaluated pressure intensities, which during threshold testing were clearly below pain threshold, as painful. This may be attributed to the different stimulation protocols used for pain quantification. While in threshold measurements the stimulation pressure increased rather quickly (increase rate, 1 kg/s), for quantification of subjective pain experience constant stimuli were applied constantly for 5 s each with 20 s interstimulus intervals. A relatively low pressure intensity delivered for 5 s may be experienced as painful even though it may be below threshold during rapid pressure increases.

Regarding the linkage between cognitive performance and pain measures, no correlations were seen for the behavioral indices of threshold and tolerance. This is in contrast to some earlier studies [13, 21–26] and might be explained by methodological differences, especially concerning the specific cognitive tests selected. However, several associations arose between cognitive parameters and VAS ratings of pain, especially those obtained during lower pressure intensities (i.e., 0.45 to 1.35 kg/cm^2^). Visual and Verbal Memory -which were respectively assessed by ROCF and TAVEC- showed more associations with cognitive performance than other cognitive measures. In fact, reproduction marks and copy execution time of ROCF as well as FPs and recognition bias of the TAVEC were associated with the majority of VAS ratings of pain.

Accuracy in the reproduction of the figure of the ROCF and planning performance on the ZMT were inversely associated with VAS scores. Concerning verbal memory, the number of FP responses and recognition bias in the TAVEC were positively associated with VAS scores. A frequently reported deficit in FMS is the slowing of cognitive processing, expressed in longer execution and reaction times [2, 5, 7, 13, 26, 43]. Moreover, higher VAS scores were associated with longer execution times in the copy condition of the ROCF, ZMT and number, letter and switching conditions of the TMT. The relationships were closer overall for low pressure intensities (especially 0.45 and 0.90 kg/cm^2^) than for the high pressure condition (2.25 kg/cm^2^). In nearly all cases, VAS ratings (regardless of pressure intensity) displayed closer associations with cognitive performance than the behavioral measures of pain threshold and tolerance. This suggests a specific role of subjective pain reports in the association with cognition in FMS. Moreover, the gradual increase of correlation coefficients with decreasing pressure underlines the closer relationship of pain responses to low stimulation intensities with cognitive impairments.

Previous studies analyzing associations between cognitive performance and clinical pain in FMS [5, 7, 16–21] have interpreted the observed inverse relationships in the context of interference effects of pain on attention and higher cognitive functions [5, 7, 11–13, 44–46]. Pain is an attention-demanding condition, which reduces the neural resources available for cognition. The brain networks underlying pain processing and attention, memory and executive functions partially overlap [2, 7, 9]. Exaggerated pain processing in FMS implies increased demands on central-nervous resources, and thus reduced resources for cognition [5, 7, 31].

However, instead of clinical pain assessment, pain intensity ratings during experimental pressure stimulation were presently obtained 2 days before cognitive testing. Therefore, it may not be appropriate to explain our results simply in terms of interference between nociceptive and cognitive processing. We hypothesize those pain responses, especially those to low stimulation intensities, reflect allodynia and central-nervous sensitization to pain. Considering this, our results may represent an association between nociceptive sensitization and cognitive performance impairments in FMS. Central-nervous sensitization in chronic pain is associated with structural and functional changes in the brain resulting from neural plasticity due to persistent nociceptive processing [47–49]. In addition to pain chronification, these processes may also affect attentional, memory and executive functions. The close associations at low stimulus intensities are also in line with evidence of perceptual amplification in FMS. Some authors have postulated that generalized hypervigilance may occur in FMS [34–35]. They referred to the Attentional Gain Control Model of Hypervigilance, according to which hypervigilant individuals experience perceptual amplification irrespective of the sensory modality [35]. FMS patients´ cognitive focus on possible painful sensations may trigger perceptual amplification involving tactile, in addition to pain, experiences, which in turn may promote hyperalgesia and allodynia [50]. This notion is also supported by findings of exaggerated somatosensory information processing in FMS and patients´ reduced capacity to habituate to repeated tactile stimulation [51–53].

In summary, this FMS study revealed closer associations of pain responses to low intensity stimulation with cognitive impairments than responses to more intense stimulation and the traditional behavioral measures of pain threshold and tolerance. Considering that pain responses to low intensity stimulation reflect hyperalgesia and allodynia, it may be hypothesized that central-nervous pain sensitization is involved in the cognitive impairments that characterize FMS. Pain reports during low intensity stimulation may be a useful measure in future studies on hypervigilance, perceptual amplification or deficits in somatosensory information processing in chronic pain.
